# Microbial community structure and the relationship with soil carbon and nitrogen in an original Korean pine forest of Changbai Mountain, China

**DOI:** 10.1186/s12866-019-1584-6

**Published:** 2019-09-13

**Authors:** Minghui Liu, Xin Sui, Yanbo Hu, Fujuan Feng

**Affiliations:** 1 College of Life Science, Northeast Forestry University, Harbin, 150040 China; 2 College of Life Science, Heilongjiang University, Harbin, 150080 China; 3 Northeast Forestry University, Harbin, 150040 China

**Keywords:** Original Korean pine forest, Soil microbial community, Biogeographic distribution, Distance-decay relationship, Soil carbon and nitrogen, Structural equation model

## Abstract

**Background:**

The broad-leaved Korean pine mixed forest is an important and typical component of a global temperate forest. Soil microbes are the main driver of biogeochemical cycling in this forest ecosystem and have complex interactions with carbon (C) and nitrogen (N) components in the soil.

**Results:**

We investigated the vertical soil microbial community structure in a primary Korean pine-broadleaved mixed forest in Changbai Mountain (from 699 to 1177 m) and analyzed the relationship between the microbial community and both C and N components in the soil. The results showed that the total phospholipid fatty acid (PLFA) of soil microbes and Gram-negative bacteria (G-), Gram-positive bacteria (G+), fungi (F), arbuscular mycorrhizal fungi (AMF), and Actinomycetes varied significantly (*p* < 0.05) at different sites (elevations). The ratio of fungal PLFAs to bacterial PLFAs (F/B) was higher at site H1, and H2. The relationship between microbial community composition and geographic distance did not show a distance-decay pattern. The coefficients of variation for bacteria were maximum among different sites (elevations). Total soil organic carbon (TOC), total nitrogen (TN), soil water content (W), and the ratio of breast-height basal area of coniferous trees to that of broad-leaved tree species (RBA) were the main contributors to the variation observed in each subgroup of microbial PLFAs. The structure equation model showed that TOC had a significant direct effect on bacterial biomass and an indirect effect upon bacterial and fungal biomass via soil readily oxidized organic carbon (ROC). No significant relationship was observed between soil N fraction and the biomass of fungi and bacteria.

**Conclusion:**

The total PLFAs (tPLFA) and PLFAs of soil microbes, including G-, G+, F, AMF, and Actinomycetes, were significantly affected by elevation. Bacteria were more sensitive to changes in elevation than other microbes. Environmental heterogeneity was the main factor affecting the geographical distribution pattern of microbial community structure. TOC, TN, W and RBA were the main driving factors for the change in soil microbial biomass. C fraction was the main factor affecting the biomass of fungi and bacteria and ROC was one of the main sources of the microbial-derived C pool.

**Electronic supplementary material:**

The online version of this article (10.1186/s12866-019-1584-6) contains supplementary material, which is available to authorized users.

## Background

Soil microorganisms, as an important component in a forest ecosystem, are sensitive to environmental change and represent the key factor affecting the decomposition of soil organic matter, nutrient cycling, and biogeochemical cycles [1, 2]. Studying the biogeochemical distribution of soil microbial communities not only helps to explore the ecological process more deeply, but also has important implications for the protection of microbial resources and the management of terrestrial forest ecosystems [3]. And increasing number of studies are showing that the composition, abundance and/or diversity of microbial communities show a certain spatial distribution pattern with some environmental variables. Nevertheless, the study of microbial biogeography is still very weak compared with that of macroorganisms (animals and plants) [4]. Whether microbial biogeographic distribution exits at different spatial scales is still controversial, particularly in terms of the distribution pattern of microbial communities along an elevation.

Soil microorganisms are the main driver for the biochemical cycle [5, 6]. Differences in spatial distribution of the soil microbial community can affect the turnover of soil carbon (C) and nitrogen (N) through its interaction with vegetation and various soil properties [7, 8]. Conversely, as important biogenic factors, the gradient effects of C and N in the soil can also cause significant effects upon the microbial community structure [9]. Obviously, the interactions between soil microorganisms and the C and N fractions are complex. Developing a deep understanding of these interactions is crucial for the sequestration and stability of C and N in the soil [8].

The broad-leaved Korean pine mixed forest (*Pinus koraiens* is as an edificator), is an important and typical component of a global temperate forest and recognized as a sensitive zone in terms of global climate change. Compared with temperate forests at the same latitude in Europe and America, the Korean pine forest in Changbai Mountain is famous for its complex structure, unique composition, and rich biological diversity. This Korean pine forest is also an important C sink for atmospheric CO_2_ [10], with an annual net ecosystem C exchange of 191.3 g/m^2^ [11]. Changbai Mountain is one of the most intact regions in the global natural ecosystem [12] and exerts great significance in regulating regional climate and maintaining regional land ecological balance. Thus, Changbai Mountain is an ideal zone with which to study the positive and negative feedback mechanisms exhibited by temperate forests upon global climate change. In particular, Changbai Mountain is one of the two main central distribution areas of natural Korean pine, where the original Korean pine broad-leaved forest remains intact at elevations of 700–1300 m.

Environmental factors (such as temperature, precipitation, and soil physicochemical properties) and the composition of vegetation vary with elevation gradient [2, 13, 14]. Changes in these factors, and their interaction, will directly, or indirectly, affect the spatial distribution patterns of soil microbial community along an elevational gradient. Our previous studies have found that soil bacterial and fungal community structures obviously varied along different elevation gradients, especially bacteria and fungi of soil showed different response by soil physicochemical properties [3, 15]. However, the distribution patterns of overall microbial taxa in this area still unknown. Investigating the distribution pattern of soil microorganisms over an elevational gradient can provide data support and a realistic basis for scientifically evaluating the biogeographical distribution of soil microorganisms [16].

In this study, we selected sites at the primary Korean pine broad-leaved forest on the northern slope of Changbai Mountain (from 699 to 1177 m) and sampled two surface soil (at depths of 0–10 cm and 10–20 cm). The aims of this study were: (1) to study the spatial distribution patterns of the soil microbial community at different elevations and explore the mechanism underlying observed differences, and (2) to analyze the relationship between the characteristics of the soil microbial community and C, N components in the soil. In carrying out this study, we expected to reveal the mechanisms of interaction between soil microorganisms and the C, N sequestration in the Korean pine forest ecosystem.

## Results

### Soil microbial community structure

**Fig. 1:**
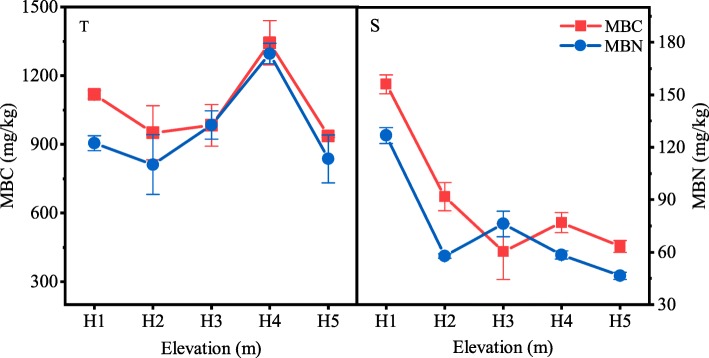
Soil microbial biomass at different elevations. MBC, microbial biomass carbon; MBN, microbial biomass nitrogen. T and S represent depths of 0–10 cm and 10–20 cm respectively

**Fig. 2:**
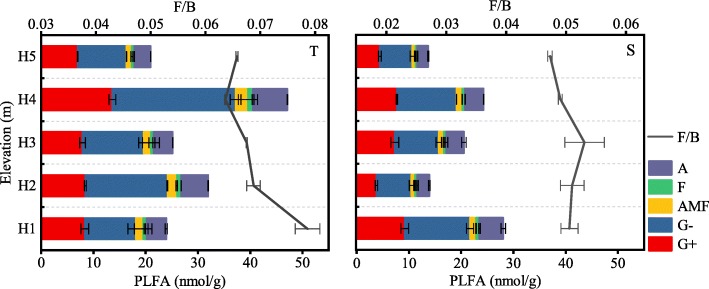
Soil microbial PLFA and Fungal/Bacterial PLFAs ratio under different elevations. T and S represent depths of 0–10 cm and 10–20 cm respectively

At a soil depth of 0–10 cm, soil microbial biomass carbon (MBC), microbial biomass nitrogen (MBN) and total PLFAs (tPLFA), Gram-negative bacteria (G-), Gram-positive bacteria (G+), fungi (F), arbuscular mycorrhizal fungi (AMF), and Actinomycetes were significantly affected by elevation gradients. All the indices were significantly different when compared across different gradients (*p* < 0.05) (Fig. 1, Fig. 2), and showed similar trends as elevation increased. All indices were highest at site H4 and lowest at site H5. In addition, The ratio of fungal PLFAs to bacterial PLFAs (F/B) ratios value were also significantly different at different sites (*p* < 0.05); the maximum values (0.062) occurred at site H1 while the minimum values (0.052) occurred at site H4 (Fig. 2).

At a soil depth of 10–20 cm, MBC, MBN, and PLFAs content, including G-, G+, F, AMF, and Actinomycetes, were significantly different at different sites. The maximum and lowest values occurred at sites H1 and H5, respectively (Fig. 1, Fig. 2). F/B ratio was both significantly different at different sites (*p* < 0.05); increasing first and then decreasing as elevation increased; the highest values were recorded at site H2 (Fig. 2).

### Biogeographical distribution of microbial communities

**Fig. 3:**
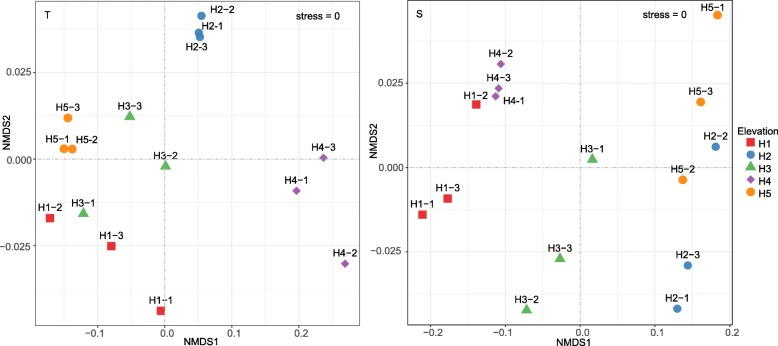
Nonmetric Multidimensional Scaling plots of soil microbial community at different elevations. T and S represent depths of 0–10 cm and 10–20 cm respectively

**Fig. 4:**
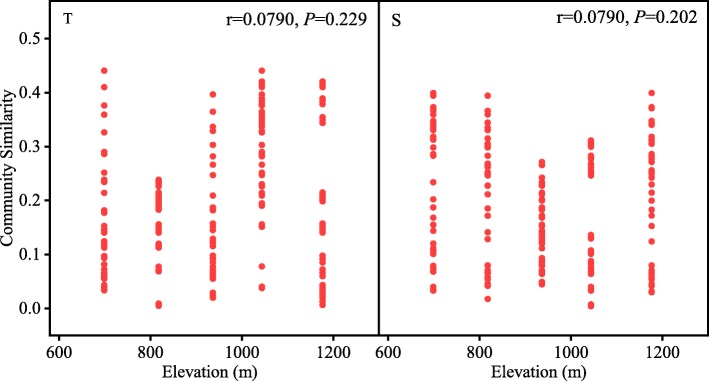
Distance-decay relationships of similarity among soil microbial communities at different elevations. T and S represent depths of 0–10 cm and 10–20 cm, respectively

Nonmetric Multidimensional Scaling plots showed that the groups of soil microorganisms at each site were clearly clustered together (Fig. 3). The relationship between microbial community composition and geographic distance did not show a distance-decay pattern (*p* > 0.05) (Fig. 4). A partial Mantel Test showed that environmental soil factors were the controlling factors affecting microbial community composition (Table 1).

**Table 1:** Relationships among dissimilarities of the microbial communities and environmental factors. Geographic distance was determined by Partial Mantel tests

Soil layer	Variables	Control for	Mantel statistics r	*p*
T	Environmental factors	Geographic distance	0.7883	0.001
	Geographic distance	Environmental factors	0.0898	0.199
S	Environmental factors	Geographic distance	0.6038	0.001
	Geographic distance	Environmental factors	0.1950	0.063

The coefficient of variation (CV) represents the degree of dispersion between data, thus reflecting the spatial differentiation of microbial groups at different elevations (sites). CV decreased in the following order: G- > Actinomycetes > AMF > G+ > F at a soil depth of 0–10 cm and G+ > Actinomycetes > G- > AMF > F at a soil depth of 10–20 cm (Table 2). Clearly, therefore, the spatial diversity of bacteria was greater than that of fungi.

**Table 2:** Variability of various microorganism PLFAs at different site

Microbial groups	T			S		
	Mean (mmol/g)	Standard deviation	Coefficients of variation (%)	Mean (mmol/g)	Standard deviation	Coefficients of variation (%)
G+	9.05	2.61	28.78	6.50	2.29	35.28
G-	14.03	5.92	42.15	9.04	2.79	30.88
AMF	1.60	0.51	31.81	0.90	0.26	28.44
F	0.70	0.20	28.30	0.51	0.11	21.54
Actinomycetes	4.43	1.41	31.83	3.16	1.01	32.10

### Controls on soil microbial community

**Fig. 5:**
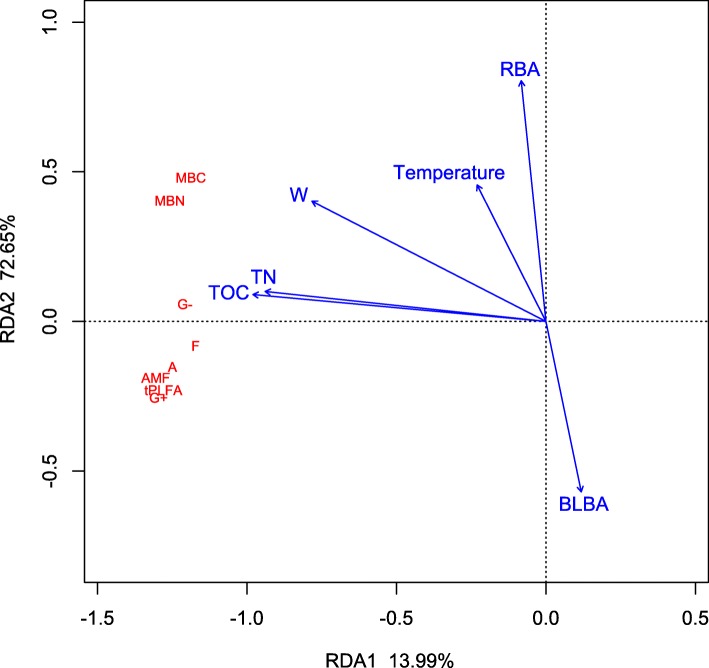
Redundancy analysis of relationships among soil microbial community structure and soil environmental factors

 Detrended correspondence analysis of the PLFAs showed that the length of the first sort axis was less than 3. Therefore, the data was analyzed by redundancy analysis based on the linear model. Then we analyzed the effects of soil physicochemical factors that might affect the microbial community, including total soil organic carbon (TOC), total nitrogen (TN), soil water content (W), temperature, pH, available nitrogen (AN), available pholsophy (AP), available K (AK), clay, silt, and sand, and the vegetation community, including the breast-height basal area of coniferous tree species per unit area (CBA), breast-height basal area of broad-leaved tree species per unit area (BLBA) and ratio of breast-height basal area of coniferous tree height to that of broad-leaved tree species (RBA), upon PLFAs of soil microbes. TOC, RBA, W, BLBA, TN, temperature were selected based on Stepwise Algorithm for RDA. As shown in Fig. 5, the first two axes of the RDA accounted for 86.64% of the variance in soil microbial community, with the first axis accounting for 72.65% of the variance. TOC (*P* < 0.001, R^2^ = 0.9141), TN (*P* < 0.001, R^2^ = 0.8435), W (*P* < 0.001, R^2^ = 0.7081), and RBA (*P* = 0.003, R^2^ = 0.3663) were the significant contributors to the variation observed in each subgroup of microbial PLFAs.

### Soil carbon and nitrogen

**Fig. 6:**
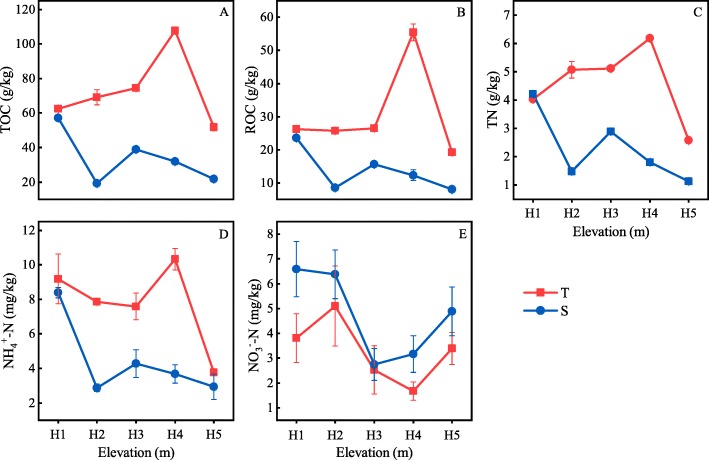
The variation of soil carbon and nitrogen with elevation. Solid lines and dotted lines represent depths of 0–10 cm and 10–20 cm respectively

Our analysis showed that soil TOC, soil readily oxidized organic carbon (ROC), TN, NH_4_^+^-N, and NO_3_^−^-N changed significantly across different sites and soil layers (*p* < 0.01). At a soil depth of 0–10 cm, TOC, ROC, TN, and NH_4_^+^-N showed similar trends of variation with increasing elevation; with maximum values at site H4 and minimum values at site H5. The NO_3_^−^-N concentration at a soil depth of 0–10 cm showed an “N”-shape changing pattern as elevation increased, with the highest value at site H2 and the lowest value at site H4, respectively (Fig. 6). At a soil depth of 10–20 cm, TOC, ROC, TN, and NH_4_^+^-N showed the highest values at site H1, with a decline with increasing elevation. The value of NO_3_^−^-N was the lowest at site H3, showing a “V”-shape changing pattern (Fig. 6).

### Relationship between soil microbial community structure and soil C and N

**Fig. 7:**
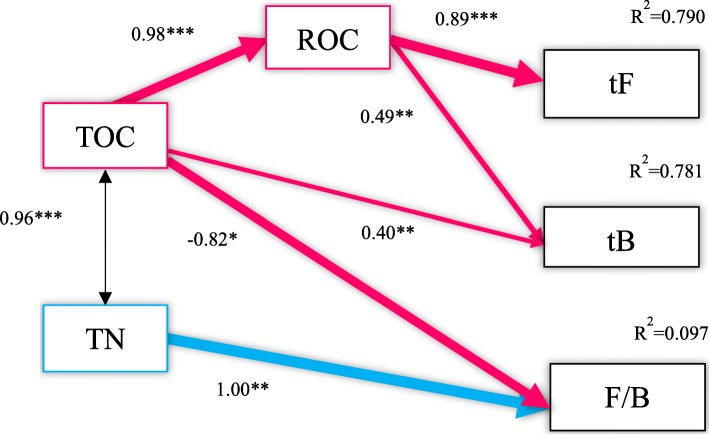
The relationships between soil microbial community and soil C, N. χ^2^ == 1.812; Df = 6; *P* = 0. 936; AIC = 31.812; GFI =0. 980; RMSEA < 0. 001; RMSEA < 0. 001. The numbers on the arrow represent the standardized path coefficient, R^2^ represents the amount of interpretation, Significant level: *, *p* < 0.05; **, *p* < 0.01; ***, *p* < 0.001; and the arrow width represents the strength of significant standardized path coefficients

The equation obtained by multiple linear regression analysis satisfied the F test (*p* < 0.05) (Table 3). Both total bacteria (tB) and total fungi (tF) were positively correlated with ROC (*p* < 0.05), which explained 78.0% and 79.0% of the changes of tB and tF, respectively. Multivariate linear regression analysis neglected the interaction between variables; in order to reflect the complex relationship within the variables more comprehensively and thoroughly, the structure equation model was used to establish the relationship between soil microbial groups and soil C and N fraction. After several fittings and revisions, we obtained the model shown in Fig. 7; the path coefficient was shown in Table 4. According to the fitting index, the model fitted well with the data. The structure equation model revealed that soil ROC had a significant correlation with the tB and tF (*p* < 0.01, *p* < 0.001), with a path coefficient of 0.49 and 0.89, respectively. The effects of soil TOC on tB were significant (*p* < 0.01), and there were indirect significant effects upon tB and tF via ROC, the indirect effect coefficients of which were 0.88 and 0.87, respectively. In addition, TOC had a significant negative correlation with F/B (*p* < 0.05). There was no significant correlation between soil TN and microbial biomass (tB, tF) (*p* > 0.05), but a significant positive correlation was observed between TN and F/B (*p* < 0.05, *p* < 0.001), with a path coefficient of 1.00.

**Table 3:** The regression analysis between soil bacterial, fungi and carbon, nitrogen fraction

Model parameter	tB(y1)			tF(y_2_)		
	(*F* = 103.908, *P* = 0.000, R^2^ = 0.780)			(*F* = 105.169, *P* = 0.000, R^2^ = 0.782)		
	coefficient	*t*	*P*	coefficient	*t*	*P*
constant	12.246	10.215	0.000	0.809	−4.163	0.000
ROC	0.721	10.194	0.000	0.047	10.255	0.000

**Table 4:** Effects of soilcarbon, nitrogen components on microbial biomass

		TOC	ROC	TN
tB	Direct effect	0.40	0.49	0.00
	Indirect effect	0.48	0.00	0.00
	Total effect	0.88	0.49	0.00
tF	Direct effect	0.00	0.89	0.00
	Indirect effect	0.87	0.00	0.00
	Total effect	0.87	0.89	0.00
F/B	Direct effect	0.82	0.00	1.00
	Indirect effect	0.00	0.00	0.00
	Total effect	−0.82	0.00	1.00

## Discussion

### Soil microbial community structure

Soil microbial tPLFA and MBC were significantly different when elevations were compared in a primitive Korean pine forest of Changbai mountain (*p* < 0.05), with a similar trend across elevations. The significant positive correlation between tPLFA and MBC (R^2^ = 0.4912, *p* < 0.05) [Additional file 2] indicated that tPLFA was a reliable indicator of biomass [17, 18]. Soil microbial tPLFA and G-, G+, F, AMF, Actinomycetes, and PLFAs showed a similar trend with changing elevation, and the content of each sub-group was significantly affected by a change in elevation (*p* < 0.05). The maximum values of the PLFA of each sub-group were found at sites H4 and H1, indicating that the microbial biomass-total PLFAs and each sub-group were the highest in the areas.

F/B, estimated by fungal PLFA and bacterial PLFA, can demonstrate timely responses to environmental changes such as aboveground vegetation and nutrient availability [2, 19]. In the present study, the value of F/B decreased significantly when the elevation increased to 1000 m (Fig. 2), indicating that the soil conditions at a higher elevation were more favorable for the survival of bacteria. Compared to fungi, bacteria prefer nutrient-rich conditions [20] which was also proven in this study through contrary vertical variations of soil C and N (Fig. 6) and F/B (Fig. 2). The F/B ratio can characterize the soil C sequestration level and stability of an ecosystem [21, 22]. Generally, an increase of F/B indicates improvements in soil C sequestration capacity and ecosystem stability [23–25]. In the present study, F/B values at sites H1 and H2 were significantly higher than those at other sites, indicating that the Korean pine forests at these sites have stronger soil C sequestration capacity and a higher stability in terms of C pools. In our previous study, the ratios of labile organic C to TOC in soils at the two sites were significantly lower than those of other sites. Both of these sets of results proved that the soils at the lower elevations (sites H1 and H2) had a higher stability in terms of the C pool and C fixation ability [26].

### Biogeographical distribution of microbial communities

The distance-decay relationship is a universal biogeographical model which reflects the spatial distribution pattern of microorganisms. This relationship is used to describe how the similarity of community composition varies with geographical distance [27]. In 1934, Baas-Becking proposed that microorganisms are small in size, large in quantity, short in generation cycle, high in diffusion rate, and that there is no dispersal limitation [28]. However, with the development of biotechnology, more and more studies are supporting the fact that the distribution pattern of microorganisms is affected by deterministic processes, or controlled by some stochastic processes, and that dispersal limitation leads to differences in community structure [29, 30]. Martiny et al. found an overall relationship between geographic distance and community similarity, but no evidence of evolutionary diversification of ammonia-oxidizing bacteria taxa at the continental scale [31]. Zhang et al. observed similar biogeographic patterns for bacterial, archaeal and methanogenic communities along the latitudinal gradient in natural wetlands and anthropogenic paddy fields [32]. In the present study, there was no distance-decay relationship in microbial communities, indicating that the spatial distribution of microbial communities was not regulated by dispersal limitation. In fact, deterministic processes of environmental heterogeneity, and stochastic processes based on diffusion constraints are widely considered as being the main factors driving microbial biogeographic distribution in a variety of environments [33–36]. One previous study showed that the relative contribution of these two processes depends upon the biogeographic-scale [31]. On small spatial scales, deterministic processes based on environmental heterogeneity were frequently reported to exert a greater impact on microbial communities than stochastic processes based on diffusion constraints [37]. Considering the relative consistency of aboveground plant groups, the spatial scale chosen in this study was small (699–1177 m), and environmental factors played a dominant role in influencing the geographical distribution pattern of the microbial community. A partial Mantel Test further demonstrated that environmental factors are indeed the most important factor for the composition of microbial species.

Han et al. [15] and Ping et al. [3] concluded that bacterial and fungal communities showed a distance-decay relationship in Korean pine forest of Changbai Mountain. In fact, this conclusion did not quantify the relationship between community similarity and geographical distance. In addition, the difference between their research and this study is that what they selected was the single microbial taxa, i.e. bacteria or fungi, rather than the whole microbial taxa. In fact, it is an indisputable fact that there are differences in the biogeographic distribution patterns of different groups of microorganisms even on the same spatial scale. It is essential to study the whole microbial taxa for a more comprehensive understanding of the microbial ecology of forest ecosystems [16].

A Nonmetric Multidimensional Scaling map showed that the microbial species composition was clearly separated at each site (Fig. 3). It was also evident that changes in elevation do have an effect on the composition of the microbial community. In both soil layers, bacteria exhibited higher CV values, indicating that bacteria were more discrete across elevations (sites). This is obviously related to the different sensitivity of microbial groups to metabolic substrates. It is generally believed that bacteria are more sensitive to soil substrates and environmental changes, whereas fungi are less sensitive [2].

### Driving factors of soil microbial community

The spatial differentiation of microorganisms can be attributed to differences in environmental conditions at different altitudes. Moreover, soil properties and spatial attributes that are associated with altitude can exert strong influences upon the composition of a microbial community [16]. Previous studies have suggested that soil TOC, TN and W are the major factors impacting upon the structure of microbial community [38, 39]. The assimilation efficiency of C and N is different among different soil microorganisms, and soil microbial community is modulated by availability and transformation of C and N [40, 41]. Soil water content can affect microbial community structure through O_2_ availability, substrate diffusion and by altering the water potential within microbial cells [42]. Redundancy analysis also showed that RBA had a significant effect upon the distribution of the soil microbiome in the primary Korean pine forest soil. This confirms our previousprediction that the proportion of coniferous and broad-leaved tree species would have a marked influence upon the soil microbial community. Researchers believed that such an effect was predominantly due to the variety and quality of litter and substrate [43]. In our study, RBA was used to characterize the differences in vegetation composition. Breast-height basal area is known to be an important indicator of the aboveground tree biomass in forest ecosystems [44], while RBA reflected the relative biomass of coniferous and broad-leaved tree species, and is directly related to the proportion of resistant organic matter for soil microbes; consequently, RBA has an important impact upon the structure of soil microbial communities [45]. The RBA at site H5 was clearly higher than at other sites. At this gradient, conifers contain more recalcitrant C, such as lignin, crude fiber, and phenolic substances, since they are difficult to be decomposed by microbes, this therefore limits the further growth of microbes. This may represent a possible factor for why the microbial biomass and distribution of microbes in this area was significantly lower than those at other sites [46]. Some studies have suggested that dominant species in forest ecosystems have profound effects on the soil environment and would have a consistent impact upon the microbial biomass in soil [47, 48]. However, we found that even if the dominant species are the same, the differences accompanying species can have an important impact on the soil microbial tPLFA and each sub-group [49].

### Relationship between soil microbial community and carbon and nitrogen

The structure equation model showed that soil ROC has a significant direct impact upon the biomass of bacteria and fungi. ROC, which is oxidized by 333 mM KMnO_4_, represents the labile organic C [50]. It is described as the “bio-available” fraction and exhibits high microbial activity and could promote the growth of fungi and bacteria [51]. Multivariate linear regression analysis also obtained similar conclusions (Table 3), indicating that soil ROC was one of the main sources of the microbial-derived C pool. TOC also had a significant indirect effect upon tB and tF through ROC, which is one of the most active components of TOC, it can be rapidly decomposed by microorganisms and provide a substrate source for its growth and activity [52]. Moreover, there was a significant negative correlation between TOC and F/B. This result indicated that the microorganisms had an effect on the stability of the soil C pool. This effect might be conferred by changing the contents of the soil TOC with a shift in community structures. High TOC could provide an advantage for bacteria to compete with fungi for resources [53]. Bacteria are dominant in the soil of *Pinus koraiensis* forest, leading to the deposition of microbial-derived C into the TOC reservoir by biomass turnover and necromass accumulation [54].

N fraction, as an important biogenic element in the process of soil microbial growth, can enhance the ability of microbes to utilize C sources, and thereby promote an increase in microbial biomass [55]. In the present study, the correlation between TN and the biomass of fungi and bacteria was not significant (Fig. 7). This result also indicated that C fraction was the main factor influencing soil fungi and bacterial biomass in the primary Korean pine forest of Changbai Mountain. We found a significant positive correlation between soil TN and F/B, and the total effect value was 1.00 (Table 4), indicating that microbes can influence N storage and N supply potential in the soil via a shift in community structure. Deng et al. further found that F/B was negatively correlated with soil N mineralization rate [8]. A low F/B is unfavorable to N accumulation in the soil, resulting in the loss of N and the reduction of N level [56]. Similar results were also obtained by de Vries et al. [57]. F/B is regarded as an important indicator for evaluating the C sequestration level in soil. In this study, we found that F/B was also closely related to soil N level.

## Conclusion

In this study, we first investigated the spatial distribution pattern of the soil microbial community across different elevations (sites) in the primary Korean pine broad-leaved mixed forest in Changbai Mountain. Our results showed that the tPLFA and PLFAs of soil microbes, including G-, G+, F, AMF, and Actinomycetes, were significantly affected by elevation. F/B ratios were significantly different in the same soil layer at different sites; F/B ratios were higher at sites H1 and H2, indicating that these areas had higher soil C sequestration capacity and C pool stability. Among the microbes investigated in each sub-group, bacteria were more sensitive to the changes of elevation. The similarities of microbial community composition did not show a distance-decay pattern, and environmental heterogeneity was the main factor affecting the geographical distribution pattern of microbial community structure. Soil TOC, TN, W and RBA were the main driving factors for the observed changes in soil microbial biomass in the Korean pine forest in Changbai Mountain. For the Korean pine forest in Changbai Mountain, soil microbial community structure and C pool stability varied with different elevations, even if the edificator was the same. Furthermore, multiple linear regression analysis and the structure equation model showed that soil ROC was one of the main sources of the microbial-derived C pool. TOC had a significant direct effect on bacterial biomass and an indirect effect upon bacterial and fungal biomass via ROC. No significant relationship was observed between soil N fraction and the biomass of fungi and bacteria; C fraction was identified as the main influential factor upon soil fungi and bacterial biomass in the original Korean pine forest of Changbai Mountain. The effect of microbes on the soil C pool may be achieved via changes in their own community structure that affect the labile organic C content in the soil. F/B showed a significant negative correlation with TOC and a significant positive correlation with TN. An increase in F/B promoted the N storage. Our results provide a theoretical basis for analyzing the in-situ ecological function of soil microorganisms in the C and N cycle of the Korean pine forest ecosystem.

## Methods

### Study area

The study was conducted at Changbai Mountain National Nature Reserve in Jilin Province, Northeastern China (41°41′49″- 42°25′18″N, 127°42′55″- 128°16′48″E). This area belongs to a temperate continental mountain climate with a long cold winter and a short cool summer. The mean annual temperature varies from 4.9 to − 7.3 °C. The annual period of sunshine is approximately 2300 h and the frost-free period is only 100 days. Annual relative humidity ranges from 65 to 74%. Precipitation is rich with a mean annual precipitation ranging from 800 and 1800 mm. The soils are dark brown forest soil. The elevation gradient and vegetation composition of each sample site are given in Additional file 1.

### Sampling

We sampled in September 2015. The method of sampling refers to Han et al. [15]. All collected soil samples were sealed and refrigerated and then taken back to the laboratory. Soil samples were then sieved through a 2-mm mesh to eliminate plant residues and roots. We then immediately determined MBC and MBN. Each homogenized soil sample was divided into two sub-samples: one was air-dried to allow the determination of the soil physicochemical properties (Table 5), including TOC, TN, and ROC, while the other sub-sample was stored at − 80 °C to allow the determination of NH_4_^+^-N, NO_3_^−^-N, and PLFA content.

**Table 5:** Soil properties of the investigated sites

Soil layer	Site	Elevation	Bulk density (BD)	Soil water content	pH	Soil temperature	Available N	Available P	Available K	Clay	Silt	Sand
(cm)		(m)	(g/cm^3^)	(%)		(°C)	(mg/kg)	(mg/kg)	(mg/kg)	(%)	(%)	(%)
T	H1	699	1.14 ± 0.09Aa	38.72 ± 6.54Aa	6.05 ± 0.01Aa	13.28 ± 3.23Aa	407.75 ± 17.6Aa	68.17 ± 5.7Aa	488.04 ± 27.3Aa	27.0 ± 1.9Aa	43.4 ± 1.2Aa	29.6 ± 0.8Aa
	H2	818	0.85 ± 0.03Ba	32.31 ± 3.89Ba	5.97 ± 0.04Ba	12.62 ± 3.10Ba	418.25 ± 22.7Ba	53.89 ± 8.6Aa	403.32 ± 13.7Ba	16.4 ± 1.3Ba	43.6 ± 0.7Aa	40.0 ± 1.4Ba
	H3	937	0.89 ± 0.09Ba	44.34 ± 5.69Ca	6.22 ± 0.03Ca	12.41 ± 3.37Ca	530.25 ± 23.3Ca	54.95 ± 6.5Ba	1027.08 ± 33.9Ca	17.8 ± 1.9BCa	48.7 ± 1.3Ba	33.5 ± 1.1Ca
	H4	1044	0.86 ± 0.05Ba	44.41 ± 6.06Ca	6.32 ± 0.01 Da	11.38 ± 3.28 Da	407.75 ± 23.9Aa	60.24 ± 7.9 Da	678.72 ± 19.4 Da	19.8 ± 1.5Ca	54.3 ± 0.9Ca	25.9 ± 1.4 Da
	H5	1177	0.99 ± 0.16ABa	28.14 ± 2.95 Da	4.95 ± 0.03Ea	10.86 ± 3.69Ea	318.50 ± 14.6Ea	52.31 ± 6.6Ea	278.88 ± 17.3Ea	17.7 ± 2.0BCa	43.1 ± 1.5Aa	39.2 ± 2.2Ba
S	H1	699	1.33 ± 0.05Ab	36.31 ± 4.70Ab	6.20 ± 0.05Aa	12.67 ± 3.45Ab	372.75 ± 15.3Ab	50.19 ± 3.4Ab	544.88 ± 18.3Ab	19.5 ± 1.1Ab	46.9 ± 1.8Ab	33.6 ± 0.8Ab
	H2	818	1.18 ± 0.00Bb	18.20 ± 6.97Bb	5.70 ± 0.07Ba	12.04 ± 3.04Bb	278.25 ± 15.8Bb	41.20 ± 2.2Bb	422.52 ± 10.5Bb	18.5 ± 1.6Aa	41.2 ± 0.7Bb	40.3 ± 2.2Ba
	H3	937	1.08 ± 0.06Cb	33.31 ± 8.76Cb	5.78 ± 0.04Ba	11.87 ± 3.53Cb	465.25 ± 20.1Cb	46.49 ± 5.60Cb	691.68 ± 21.5Cb	18.2 ± 0.8ACa	45.8 ± 1.3Aa	36.0 ± 0.8Ab
	H4	1044	1.31 ± 0.05Ab	16.74 ± 4.04Db	6.13 ± 0.09Aa	10.93 ± 3.28Db	276.50 ± 11.2Bb	42.79 ± 4.1Db	252.96 ± 11.6Db	11.8 ± 1.7Bb	38.2 ± 2.1Cb	50.0 ± 1.8Cb
	H5	1177	1.17 ± 0.07Bb	17.02 ± 2.07Db	5.16 ± 0.13Ca	10.47 ± 4.07Eb	162.75 ± 10.7Eb	17.41 ± 2.7Eb	164.64 ± 14.8Eb	15.0 ± 2.9BCa	37.4 ± 1.8Cb	47.6 ± 1.2Cb

T and S represent depths of 0–10 cm and 10–20 cm respectively. Different capital letters in the same column represent significant differences among different elevation gradients at the 0.05 level. Different lowercase letters in the same column represent significant differences among different layers at the 0.05 level. Soil water content and Temperature were the average of the whole of grow season (May-Oct).

### Composition of tree species in experimental areas

Within the sample plots at each site, we recorded the different types of each living tree species present and determined the diameter at breast height (DBH) (≥ 5 cm) for each tree. The CBA (Eq. 1), BLBA (Eq. 2) and RBA (Eq. 3) were also calculated. The derivation of these parameters is shown below.

1 $$ \mathrm{CBA}=\uppi \times {\left(\frac{{\mathrm{DBH}}_{\mathrm{CBA}}}{2}\right)}^2 $$

2 $$ \mathrm{BLBA}=\uppi \times {\left(\frac{{\mathrm{DBH}}_{\mathrm{BLBA}}}{2}\right)}^2 $$

3 $$ \mathrm{RBA}=\frac{\mathrm{CBA}}{\mathrm{BLBA}} $$

### Analysis of C and N components in soil samples

TN and TOC were measured in soil samples by a EuroEA3000 element analyzer (Leeman company, America) while ROC was determined by 333 mM KMnO_4_ oxidation [50]. In brief, air-dried soil, containing approximately 15 mg of C, was placed in a 50-mL centrifuge tube and 25 mL of 0.333 M KMnO_4_ solution was added; the mixture was then shaken at 120 *rpm* for 1 h. After centrifugation, the supernatant was diluted by a factor of 1:250 with sterile water. A blank experiment, without soil, was treated in a similar manner. The absorbance of the diluted solution was then measured at 565 nm, and the ROC was then calculated according to the absorbance. NH_4_^+^-N was analyzed using the indophenol blue colorimetric method. Fresh soil samples were extracted with 2 M KCl solution. Then, 10 mL of the extract was mixed with 5 mL of phenol solution, and 5 mL of sodium hypochlorite alkaline solution at room temperature for 1 h. The NH_4_^+^-N concentration in the extracted solutions was then determined at an absorbance of 625 nm. NO_3_^−^-N was determined by the phenol disulfonic acid method. In brief, fresh soil samples were extracted with distilled water and then evaporated until they were fully dry. Then, 2 mL of phenol disulfonic acid reagent was added quickly and incubated for 10 min, followed by 20 mL of distilled water and excess (1:1) of ammonium hydroxide. The absorbance of the solution was then measured at a wavelength of 420 nm. MBC and MBN were estimated using the fumigation-extraction method [58]. In brief, fresh soil samples were fumigated with ethanol-free chloroform for 24 h in the dark at 25 °C. Fumigated soil was then extracted with K_2_SO_4_ solution; non-fumigated soil was treated in the same way. MBC and MBN were then determined using an Elementar Vario Max element analyzer (Elementar, Germany) according to the differences in C and N content between fumigated soil and non-fumigated soil with an internal conversion coefficient of 0.45.

### Analysis of phospholipid fatty acids in soil samples

Soil microbial community structure was analyzed using PLFAs as described previously [59]. In brief, 8 g of fresh soil was extracted with a liquid mixture of chloroform: methanol: phosphate buffer (1:2:0.8 by volume) for 2 h. After centrifugation, the supernatant was transferred to a separatory funnel and mixed with 12 mL of chloroform and 12 mL of phosphate buffer for 2 min. The lower phase was then collected and extracted with 23 mL of the liquid mixture for 30 min. After centrifugation, the separated mixture was allowed to rest overnight. The lower phase was then concentrated by N_2_ at a temperature of 30–32 °C and used in lipid fractionation. The concentrated lipid extract was dissolved in 200 μL of chloroform and fractionated from other lipids on solid phase extraction columns (Supelco Inc., Bellefonte, PA). Neutral and glycol lipids were eluted by 5 mL of CHCl_3_ and 10 mL of acetone; polar lipids were then eluted by 5 mL of methanol, respectively. The methanol phase was then collected and dried under N_2_ at a temperature of 30–32 °C. The dried lipids were then mixed with 1 mL of a 1:1 solution of methanol and toluene and 1 mL of 0.2 mol/L potassium hydroxide, heated at 37 °C for 15 min, then dried under N_2_ after extraction with n-hexane. Finally, fatty acid methyl esters were detected by gas chromatography-mass spectrometry (Agilent 6850 series Gas Chromatograph) equipped with an HP-5 capillary column (25.0 m × 200 mm × 0.33 mm) with N_2_ as the carrier gas. Concentrations of each PLFA were calculated based on the 19:0 internal standard concentrations and abundances were expressed as nmol per gram dry soil. The PLFAs used as biomarkers are shown in Table 6. F, AMF were generally attributed to tF [65], and tB was calculated as the sum of G+, G-, and Actinomycetes biomarkers together with 10:0; 13:0; 16:0, and 20:0 [59, 66]. PLFAs not assigned as biomarkers were included in total PLFA (tPLFA) yields.

**Table 6:** Phospholipid fatty acids (PLFAs) used as biomarkers

Microbial group	Fattyacidtype	Phospholipids fatty acid signatures	Reference
Gram-positive bacteria	a−/I-branched fatty acids	12:0 iso, 13:0 iso, 13:0 anteiso, 14:0 iso, 15:0 iso, 15:0 anteiso, 16:0 iso, 16:0 anteiso, 17:0 iso, 17:0 anteiso, 18:0 iso, 19:0 iso, 19:0 anteiso, 20:0 iso	[60, 61]
Gram-negative bacteria	monounsaturated fatty acids and cyclopropane fatty acids	11:0; 10:0 2OH, 2OH 12:0;3OH i14:0;14:1 w5c, 15:1 w5c,15:1 w6c, 16:1 w9c, 16:1 w7c, 16:0 2OH, 17:1 w8c, 17:1 w7c, 18:1 w7c, 18:1 w5c, 20:1 w9c, 3OH 12:0; 2OH 13:0; 3OH i12:0; 3OH i15:0; 3OH i16:0; 3OH i17:0; 12:0; 14:0, 15:0, 17:0,	[2, 61, 62]
Actinomycetes	Methyl branched fatty acids	16:0 10-methyl, 17:0 10-methyl, 18:0 10-methyl	[60]
Fungi		18:2 w6c, 18:2w9c	[63]
Arbuscular mycorrhizal fungi		16:1 w5c	[64]

### Statistical analyses

One-way analysis of variance was used to determine the effects of elevations (sites) on soil physicochemical properties, C and N fractions, microbial biomass, and microbial PLFAs. The significance level was set to *p* < 0.05. Statistical analysis was conducted in SPSS 19.0 for Windows. Nonmetric Multidimensional Scaling was used to visualize the overall differences in microbial community dissimilarities across different elevations (sites)(www.omicshare.com/tools/). Using R studio, we used the Mantel test and the Partial Mantel test, to determine correlations between environmental variability and geographic distance and the variation of microbial community. Ordination was used to analyze the relationship between the composition of soil microbial community and soil environmental factors. Detrended correspondence analysis was performed for microbial PLFAs to determine whether the community was ranked by a unimodal model or a linear model. The statistical significance of the redundancy analysis was tested using the Monte Carlo permutation test (499 permutations; *p* < 0.05).

The relationships between microbial biomass, microbial community structure, and the C and N components in soil were analyzed using multivariate linear regression analysis by SPSS 19.0 and the structure equation model by Amos 21.0 software. Multivariate linear regression analysis was performed stepwise method which selected tB, tF and F/B as dependent variables and soil TOC, ROC, TN, NH_4_^+^-N and NO_3_^−^-N as independent variables. We started a priori model based on a literature review and our knowledges. The utilization and transformation of organic C and N by microorganisms is generally thought to mainly include the dominant pathways of fungi and bacteria [67]. Therefore, the fungal biomass and bacterial (tF, tB) and F/B were selected to represent indices of the soil microbial community structure. Moreover, soil labile organic C and inorganic N were considered to provide C source and N source for microbial metabolization, respectively, which may impact upon microbial biomass [68, 69]. Previous studies have also found that the ratio of fungi to bacteria may affect the storage of soil C and N [8]. Then, the relationship between soil microbial community structure and soil C and N components was established. In order to get the most parsimonious model, the paths and indicators which were not significant were deleted. The adequacy of the model was determined by non-significant χ^2^-tests(*P* > 0.05), goodness-of-fit (GFI ≥ 0.90) and root square mean error of approximation (RMSEM< 0.1). Moreover, Akaike information criterion (AIC) was used to determine the model that was the most parsimonious with the data [70].

## Additional files

Additional file 1: Vegetation distribution of the sampling areas. (DOCX 19 kb)

Additional file 2: Correction between tPLFA and MBC. (PNG 13 kb)

## Data Availability

All data generated or analyzed during this study are included in this published article and its supplementary information files. The raw data are available from the corresponding author on reasonable request.

## Abbreviations

AIC: Akaike information criterion
AMF: Arbuscular mycorrhizal fungi
BLBA: Breast-height basal area of broad-leaved tree species per unit area
C: Carbon
CBA: Breast-height basal area of coniferous trees species per unit area
F: Fungi
F/B: Ratio of fungal PLFAs to bacterial PLFAs
G-: Gram-negative bacteria
G+: Gram-positive bacteria
GFI: Goodness-of-fit
MBC: Microbial biomass carbon
MBN: Microbial biomass nitrogen
N: Nitrogen
PLFA: Phospholipid fatty acid
RBA: Ratio of breast-height basal area of coniferous trees to that of broad-leaved tree species
RMSEM: Root square mean error of approximation
ROC: Readily oxidized organic carbon
tB: Bacterial biomass
tF: Fungal biomass
TN: Total nitrogen
TOC: Total organic carbon
tPLFA: Total PLFAs
W: Soil water content
