# Supplementary material for: Microbial community structure and the relationship with soil carbon and nitrogen in an original Korean pine forest of Changbai Mountain, China
Source: BMC Microbiol. 2019 Sep 13;19:218. doi: 10.1186/s12866-019-1584-6 (PMC6743161; doi:10.1186/s12866-019-1584-6)
Supplement: Supplementary file 1 — Vegetation distribution of the sampling areas. (DOCX 19 kb) [file 12866_2019_1584_MOESM1_ESM.docx]

Table A. 1 Vegetation distribution of the sampling areas

| Elevation  (m) | Latitude and longitude | Major plant species | | | Average height  (m) | Canopy density | CBA (cm^2^) | BLBA (cm^2^) | RBA |
| --- | --- | --- | --- | --- | --- | --- | --- | --- | --- |
|  |  | Arbor | Shrub | Herbal |  |  |  |  |  |
| 699 | 42°24′42″N, 128°05′48″E | *Pinus koraiensis*, *Acer mono*, *Tiliaamurensis*, *Fraxinusmandshurica*, *Quercus mongolic*a, *Populusussuriensis*, *Acer triflorum*, *Syringa reticulata* | *Philadelphusincanus*, *Sorbariasorbifolia*, *Lonicera japonica*, *Ribesnigrum*, *Viburnum sargenti* | *Brachybotrysparidiformis*, *Smilacina japonica*, *Athyriumbrevifrons*, *Thalictrum aquilegifolium*, *Hylomecon japonica*, *Geranium wilfordii*, *Hibiscus trionum* | 15.5 | 0.7 | 21.69±6.5AB | 25.91±2.1AB | 0.8±0.3A |
| 818 | 42°21′09″N, 128°05′48″E | *Pinus koraiensis*, *Betula platyphylla*, *Tiliaamurensis*, *Fraxinusmandshurica*, *Abiesnephrolepis*, *Phellodendronamurense*, *Piceakoraiensis*, *Populuskoreana*, *Syringa reticulata* | *Philadelphusincanus*, *Spiraeasalicifolia*, *Rosa davurica*, *Rubuscrataegifoliu*, *Euonymus phellomana*, *Corylus mandshurica*, *Lonicera ruprechtiana*, *Viburnum sargenti*, *Euonymus alatus*, *Acanthopanaxsenticosus* | *Equisetum hyemale*, *Lamiumbarbatum*, *Brachybotrysparidiformis*, *Geranium wilfordii*, *Paraseneciohastatus*, *Solanum nigrum*, *Potentillacryptotaeniae* | 15.3 | 0.7 | 8.63±0.86A | 25.48±5.9AC | 0.4±0.2A |
| 937 | 42°17′50″N, 128°08′11″E | *Pinus koraiensis*, *Tiliaamurensis, Quercus mongolic*a, *Abiesnephrolepis*, *Piceajezoensis*, *Acer mandshuricum*, *Acer mono*, *Ulmuslaciniata*, *Larixgmelinii*, *B．costata*, *Populusdavidiana*, *Acer tegmentosum*, *Acer mono* | *Acanthopanaxsenticosus*, *Sorbariasorbifolia*, *Sambucuswilliamsii*, *Spiraeasalicifolia*, *Viburnum sargenti*, *Rubuscrataegifolius*, *Lonicera japonica* | *Cirsium japonicum*, *Lamiumbarbatum*, *Geranium wilfordii*, *Dryopteriscrassirhizoma*, *Brachybotrys paridiformis Maxim. ex Oliv. Brachybotrysparidiformis*, 0.8*RhizomaParidis*, *Equisetum hyemale*, *Thalictrum aquilegifolium* | 15.6 | 0.8 | 22.24±10.43AB | 16.71±7.05BC | 1.9±2.0A |
| 1044 | 42°14′07″N, 128°09′34″E | *Pinus koraiensis*, *Piceajezoensis*, *Piceakoraiensis*, *Abiesnephrolepis*, *Acer mono*, *Tiliaamurensis*, *Fraxinusmandshurica*, *Populuskoreana*, *Populuscathayana*, *Larixgmelinii*, *Acer tegmentosum* | *Euonymus alatus*, *Acanthopanaxsenticosus*, *Ribesmandshuricum*, *Rosa davurica* | *Paraseneciohastatus*, *Brachybotrysparidiformis*, *Dryopteriscrassirhizoma*, *Equisetum hyemale*, *RhizomaParidis*, *Athyriumbrevifrons* | 16.8 | 0.8 | 28.51±11.12B | 42.07±22.7A | 1.2±1.3A |
| 1177 | 42°10′52″N, 128°09′35″E | *Pinus koraiensis*, *Abiesnephrolepis*, *Piceajezoensis*, *Larixolgensis*, *Piceakoraiensis*, *Betula platyphylla* | *Sorbariasorbifolia*, *Acanthopanaxsenticosus*, *Euonymus alatus* | *Equisetum hyemale* | 16.6 | 0.8 | 31.82±4.44B | 5.77±1.31C | 5.6±0.7B |

CBA: breast-height basal area of coniferous trees species per unit area; BLBA: breast-height basal area of broad-leaved tree species per unit area; RBA: ratio of breast-height basal area of coniferous tree height to that of broad-leaved tree species; The measurement and calculation methods of the ratio of [breast-height basal area](http://dict.cnki.net/dict_result.aspx?searchword=%e8%83%b8%e9%ab%98%e6%96%ad%e9%9d%a2%e7%a7%af&tjType=sentence&style=&t=breast-height+basal+area) of coniferous and broad-leaved trees are described in Section 1.3.
