# Supplementary figures and images for: Microbial community structure and the relationship with soil carbon and nitrogen in an original Korean pine forest of Changbai Mountain, China
Source: BMC Microbiol. 2019 Sep 13;19:218. doi: 10.1186/s12866-019-1584-6 (PMC6743161; doi:10.1186/s12866-019-1584-6)

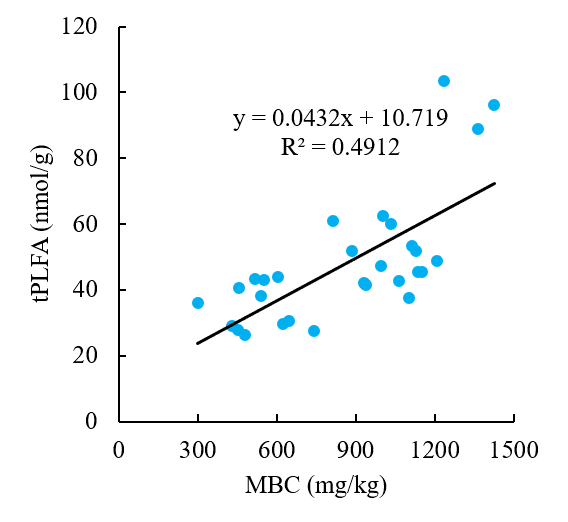

Supplement: Supplementary file 2 — Correction between tPLFA and MBC. (PNG 13 kb) [file 12866_2019_1584_MOESM2_ESM.png]
